# Pseudorabies in pig industry of China: Epidemiology in pigs and practitioner awareness

**DOI:** 10.3389/fvets.2022.973450

**Published:** 2022-09-16

**Authors:** Lei Tan, Yujun Zhou, Yixing Qiu, Lei Lei, Cheng Wang, Pei Zhu, Deyong Duan, Hongyu Lei, Lincheng Yang, Naidong Wang, Yi Yang, Jun Yao, Wei Wang, Aibing Wang

**Affiliations:** ^1^Lab of Animal Disease Prevention and Control and Animal Model, Hunan Provincial Key Laboratory of Protein Engineering in Animal Vaccines, College of Veterinary Medicine, Hunan Agricultural University (HUNAU), Changsha, China; ^2^Hunan Sino-science Gene Technology Co., Ltd, Changsha, China; ^3^TCM and Ethnomedicine Innovation and Development International Laboratory, Academician Atta-ur-Rahman Belt and Road Traditional Medicine Research Center, School of Pharmacy, Hunan University of Chinese Medicine, Changsha, China; ^4^Xiangxi Prefecture Animal Husbandry and Aquatic Products Affairs Center, Xiangxi, China; ^5^Yunnan Tropical and Subtropical Animal Virus Diseases Laboratory, Yunnan Animal Science and Veterinary Institute, Kunming, China; ^6^PCB Biotechnology LLC, Rockville, MD, United States

**Keywords:** pseudorabies virus, porcine industry related practitioners, questionnaire survey, China, epidemiology

## Abstract

Pseudorabies virus (PRV) is widely prevalent in China, which can transmit from pigs to other mammals. Moreover, a PRV variant isolated from an acute human encephalitis case was documented recently. It is imperative to investigate PRV epidemiology in pigs, the knowledge regarding pseudorabies (PR) and self-protection behaviors upon working among relevant practitioners including pig farmers, pig cutters, and pork salesman. In the present study, 18,812 pig serum samples and 1,634 tissue samples were collected from Hunan Province during the period of 2020 to 2021 for detecting the presence of PRV gE-special antibody and nucleic acids, respectively. Meanwhile, we conducted a questionnaire survey about PR among these practitioners in China. The results showed that nearly 9% (1,840/20,192) pigs from 161 collected sites (20.17%, 161/797) were seropositive for PRV-gE antibody. Though only 2.33% tissue samples were positive for PRV nucleic acids, all the representative PRV strains were variant. It was learned that most practitioners were frequently injured when working, the injured sites mainly included hand and foot. Among the three transmission routes of PRV, the aerosol transmission route was often overlooked. Moreover, the workers lacked self-protection awareness and were poor conscious about PRV and its potential threat to humans. All the results demonstrate that PRV remains widely spread in pig populations, while the potential threats of PRV in pig industry receive less attention, suggesting that targeted educational programs to these people should be performed.

## Introduction

Pseudorabies virus (PRV) is the causative agent of pseudorabies (PR) and belongs to the *Alphaherpesvirus* subfamily of the *Herpesviruses* (1). Pigs are the unique natural hosts for PRV infection, the clinical symptoms of PR mainly include severe neurological symptoms (tremor, dyskinesia, and lethargy, etc.,) and reproductive disorders (2). Besides pigs, a variety of mammals (such as ruminants, rodents, and canines) are also susceptible to PRV, the disease (PR) is always fatal to these non-natural hosts and mainly characterized by severe pruritus and neurological symptoms (3).

Whether PRV naturally infects humans has been a controversial topic since the initial document of this pathogen in 1902 (4). However, the problem did not really come to the fore until increasing human encephalitis cases caused by PRV infection were documented in China (5–9). The clinical signs of PR in humans were primarily characterized by fever, headache, and encephalitis, etc., even the patients would die without effective and timely treatments (3). Remarkably, most of patients in these reported cases were porcine industry related workers, such as pig farmers, pig cutters, pig butchers, and veterinarian. Therefore, it is necessary to carry out relevant investigations.

This study was aimed to investigate the epidemiological characteristics of PRV among pig populations in Hunan Province of China, and evaluate the awareness and behavior concerning the emerging PRV infection among pig farmers, pig cutters, and pork salesman in China, who are at high risks of being exposed to this infectious virus.

## Methods and materials

### Investigation areas and questionnaire design

The cross-sectional investigation was mainly conducted in Hunan and Yunnan Provinces of China during autumn and winter in 2021 (Supplementary Figure S1). These two provinces were selected for their high-density of pig breeding. More importantly, one human case with encephalitis and endophthalmitis caused by PRV infection was recently reported in Hunan Province (10).

Two questionnaires were developed to evaluate the knowledge level concerning PRV among pig cutters and pork salesman, pig farmers, respectively. The questionnaires mainly included items about characteristics of participants (such as gender, age, education level, and working years) (Supplementary Table S1), self-protection upon working (e.g., Have you ever got injured upon working? Where are the injured sites? and What's your self-protection measures upon working?) (Table 1), and awareness of PRV and its potential risk to human's health (e.g., “Have you ever heard about PRV? “Does PRV infect other mammals except pigs?” and Do you think PRV pose a huge threat to human's heath?”) (Table 2), etc.

**Table 1:** Injury and self-protection upon working among pig-related practitioners.

**Questionnaires**	**Pig farmers (%)**	**Pig cutters (%)**	**Pig salesmen (%)**
**Have you ever got injured upon working?**			
Never	10.97 (34/310)	3.11(5/161)	6.25 (4/64)
Occasionally	78.06 (242/310)	81.98 (132/161)	85.94 (5/64)
Often	10.97 (34/310)	14.91 (24/161)	7.81 (5/64)
**Where is the injured position (s) upon working?**			
Hand	83.70 (231/276)	94.23 (147/156)	90.0 (54/60)
Arm	26.45 (73/276)	7.09 (12/156)	5.0 (3/60)
Leg	48.55 (134/276)	10.26 (16/156)	6.67 (4/60)
Head or face	6.52 (18/276)	4.49 (7/156)	3.33 (2/60)
Other positions	18.16 (50/276)	8.33 (13/156)	6.67 (4/60)
**Do you treat with the wound after injury?**			
Small wounds, Yes	64.86 (179/276)	81.41 (127/156)	71.67 (43/60)
Small wounds, No	35.14 (94/276)	18.59 (29/156)	28.33 (17/60)
Big wounds, Yes	98.55 (272/276)	100.0 (156/156)	96.67 (58/60)
Big wounds, No	1.45 (4/276)	NA	3.33 (2/60)
**What is your self-protection measure upon working?**			
None	29.68 (92/310)	1.86 (3/161)	46.88 (30/64)
Wear gloves	20.0 (62/310)	14.29 (23/161)	7.81 (5/64)
Wear a mask	39.35 (122/310)	39.13 (63/161)	29.69 (19/64)
Wear gloves and mask	10.97 (34/310)	44.72 (72/161)	15.62 (10/64)

**Table 2:** General knowledge about PRV among pig-related practitioners.

**Questionnaires**	**Pig farmers (%)**	**Pig cutters (%)**	**Pig salesmen (%)**
**Have you ever heard of PRV?**			
Yes	98.71 (306/310)	62.11 (100/161)	23.44 (15/64)
No	1.29 (4/310)	37.89 (61/161)	76.56 (49/64)
**Knowledge of the transmission route of PRV**			
Vertical transmission	75.16 (233/310)	32.30 (52/161)	10.94 (7/64)
Horizontal transmission	85.16 (264/310)	48.45 (78/161)	7.81 (5/64)
Aerosol transmission	40.0 (124/310)	13.66 (22/161)	3.13 (2/64)
**Knowledge of susceptible species to PRV**			
Only pigs	16.77 (52/310)	44.10 (71/161)	20.31 (13/64)
Pigs and other animals	31.29 (97/310)	4.35 (7/161)	3.13 (2/64)
Pigs, other animals, and humans	51.94 (161/310)	13.66 (22/161)	NA
I do not know	NA	37.89 (61/161)	76.56 (49/64)
**Knowledge of the major clinical signs of PRV infection in humans**			
Fever, encephalitis, dyspnea, etc.,	44.01% (136/310)	10.56 (17/161)	NA
I do not know	55.99% (173/310)	89.44 (144/161)	100 (64/64)
**Knowledge of possible routes of PRV transmission from pig to humans**			
Only respiratory tract	4.85 (15/310)	4.97 (8/161)	3.13 (2/64)
Only wound or eye	30.74 (95/310)	45.97 (74/161)	57.81 (37/64)
Respiratory tract, wound, or eye	53.07 (164/310)	37.89 (61/161)	26.56 (17/64)
I do not know	11.33% (35/310)	11.18 (18/161)	12.5 (8/64)

### Participants selection

For the interview of different participants, 1–4 pig farmers with different works (e.g., breeder, executive director, veterinarian) were selected in one pig farm according to its breeding scale. 2–4 pig cutters were selected in each slaughterhouse, and one pork salesman whose works include segmentation and sale was selected from each stall in wet market or supermarket. All interviewers were informed about this study and their consent was obtained before conducting the questionnaire survey. The questionnaire was performed in a face-to-face or on-line way, which needed nearly 10 min to complete.

### Serum specimen collection and serological detection

From 2020 to 2021, 18,812 blood specimens were collected from pigs at different development stages among 798 collection sites (including pig farms and slaughterhouses) from Hunan Province of China. The blood samples were labeled with essential information (regions and collection dates), and centrifuged (3,000 rpm, 4°C) for 15 min. Subsequently, the sera were collected, transferred to labeled cryotube, and stored at −20°C for further procession.

The presence of anti-gE antibodies among 18,812 serum samples was detected by using a commercial blocking ELISA kit (Cat: CP144, IDEXX Laboratories, Inc. Westbrool, ME) according to the manufacturer's instructions, which could differentiate between the naturally PRV-infected and vaccinated pigs.

### Clinical sample collection and molecular detection

Tissue samples (lymph node, lung, brain, etc.) were collected from 1,634 PR-suspected pigs with clinical symptoms of encephalitis, diarrhea, fever (piglets), reproductive disorders (sows). Viral genomic DNAs were extracted from each sample using commercial kits (Genenode Biotech Co.Ltd., China) following the manufacturer's instructions.

PCR was performed to detect the presence of PRV nucleic acids as previously described (11). Moreover, some positive PCR products were cloned, sequenced, and analyzed as previously described (12).

### Data analysis

All data were input into the Microsoft Excel 2010 software (Microsoft, Redmond, WA, USA) after each questionnaire survey. The significance of potential correlations between the PRV positive rate and other factors including year and season were analyzed using Chi-square test in SPSS 20.0 software (IBM, Chicago, IL, USA). A *p*-value <0.05 was considered statistically significant.

## Results

### Seroprevalence of PRV-gE in Hunan Province

As shown in Table 3, at the collection site level, the proportion of PRV-positive collected sites was 20.17% (161/798), and the proportions of which in 2020 and 2021 were 23.51% (87/370) and 17.29% (74/428), respectively (*P* < 0.05). Among different seasons, the proportions of PRV-positive collected sites were 18.39% (48/261), 23.21% (52/224), 13.98% (26/186), and 29.13% (37/127) in spring, summer, autumn, and winter, respectively (*P* < 0.05). At the sample level, 1,667 (8.91%) serum samples were sero-positive for PRV-gE antibody. Similar with the results of collection site level, the positive rate of PRV-gE antibody in serum samples collected from autumn was significantly lower than which from winter (*P* < 0.05).

**Table 3:** Seroprevalence of PRV-gE in pig populations in Hunan Province during 2021–2021.

**Year**	**Season**	**No. collected sites**	**No. positive sites**	**Positive rate (%)**	**95% CI**	***p***	**No. samples**	**No. positive samples**	**Positive rate (%)**	**95% CI**	***p***
2020	Spring	70	22	34.42	23.29–45.55	>0.05	1,360	149	10.96	9.30–12.62	<0.05
	Summer	118	26	22.03	14.55–29.51	>0.05	3,498	339	9.69	8.71–10.67	<0.05
	Autumn	136	23	16.91	10.61–23.21	Reference	3,520	270	7.67	6.79–8.55	Reference
	Winter	46	16	34.78	21.02–48.54	>0.05	1,168	133	11.37	9.55–13.19	<0.05
Subtotal		370	87	23.51	19.19–27.83	<0.05	9,546	891	9.33	8.75–9.91	<0.05
2021	Spring	191	26	13.61	8.75–18.47	<0.05	5,008	335	6.68	5.99–7.37	<0.05
	Summer	106	26	24.52	16.33–32.71	<0.05	2,281	132	5.78	4.82–6.74	>0.05
	Autumn	50	3	6.0	0–12.58	Reference	812	30	3.69	2.39–4.99	Reference
	Winter	81	19	23.46	14.23–32.69	<0.05	1,165	289	24.81	22.33–27.29	<0.05
Subtotal		428	74	17.29	13.71–20.87	Reference	9,266	786	8.48	7.91–9.05	Reference
Total		798	161	20.17	17.39–22.95		18,812	1,677	8.91	8.50–9.32	

The periods of spring, summer, autumn, and winter were March–May, June–August, September–November, January–February and December, respectively.

### PRV detection and sequence homology analysis

Among the 1,634 tissue samples collected for PRV investigation by PCR, 38 samples (2.33%) were PRV-positive. The positive rate of PRV among tissue samples in 2021 was slightly higher than that in 2020, with 2.5% (25/1,020) for 2021 and 2.1% for 2020 (13/614), respectively. Nine PRV-positive PCR products were selected for further phylogenetic analysis, which showed that all of them were identified as PRV variants (data not shown).

### Participant characteristics

As shown in Supplementary Table S1, 535 participants (310 pig farmers, 161 pig cutters, and 64 pig salesman) were included in this study. Most of respondents (70.28%, 376/535) were male, the others (29.72%, 159/535) were female. The majority (73.83%, 395/535) completed their second or high school education, a few participants (4.11%, 22/535) only received primary school or no education. The others (22.06%, 118/535) in pig farms and cutter groups obtained their bachelor or master's degree, even doctoral degree.

Among 310 interviewed pig farmers, PR has been eradicated in nearly 60% (189/310) pig farms where the participants worked, only 57 (18.45%) respondents declared PRV was being prevalent in their worked farms. In spite of these, over 96% (298/310) participants stated that pigs in their farms were routinely immunized with PRV vaccines, and nearly half of these pig farms chose attenuated live vaccines. Most of participants (88.06%, 273/310) still believed that PRV was severely prevalent in China.

### Injury and self-protection upon working among different participants

In total, 78.26% of pig farmers, 81.98% of pig cutters, and 85.94% of pig salesmen reported to occasionally get injured upon working. While a minority of respondents said that they often got injury upon working, with the highest rate reported by pig cutters (14.91%, 24/161). The injury positions mainly included hand, leg, and arm, however a few participants reported that they ever got injury on the head or face. As far as the wound treatment concerned, 70.93% (349/492) interviewers said they took no treatment measure to small wounds, such as scratch or small broken skin, but nearly all of them claimed that they adopted almost immediate treatment to serious injury. Ninety-two pig farmers (29.68%) and 30 pig salesmen (46.88%) asserted that they worked without any self-protection measures, only a small group of pig farmers and pig salesmen expressed that they would wear a mask and gloves when working (Table 1).

### Basic knowledge about PRV among different participants

Of 310 pig farmers, 161 pig cutters, and 61 pig salesmen, 1.29%, 37.89%, and 76.56% respectively never heard of PRV. Most of the participants focused on vertical and horizontal transmission routes, while the aerosol transmission route was always neglected. Moreover, nearly half of pig farmers did not know that PRV could infect humans except for pigs and other animals, as compared to 86.34% (139/161) of pig cutters and all of salesmen. Nearly 60% of pig farmers, 90% of pig cutters, and all pig salesmen did not know the clinical signs of PRV infection in humans. In addition, 53.07% (164/310) of pig farmers correctly answered the possible routes of PRV transmission in humans, while the majority of pig cutters (62.11%, 61/161) and salesmen (73.44%, 47/64) wrongly answered it (Table 2).

### Concerns of participants regarding zoonotic diseases (e.g., PRV infection)

Many respondents expressed their concerns regarding the potential threats of zoonotic diseases, Among 535 participants, 207 (66.77%) pig farmers, 25 (15.53%) pig cutters, and 7 (10.94%) pig salesmen agreed that pigs or pork were at high risk of being infected/contaminated with zoonotic pathogens, and nearly 40% respondents worried that some zoonotic pathogens could be transmitted from pigs/pork to humans. Considering the threats of PRV to humans, more than half of pig farmers (56.78%, 176/310) thought the presence of an exposure risk to this infectious virus at work, especially the application of attenuated vaccines of pigs. On the contrary, the majority of pig cutters (80.74%, 130/161) and salesmen (75.0%, 48/64) disagreed with it. As for measures to prevent PRV infection at work, almost all participants hoped to timely deal with wounds and actively learn relevant knowledge, 198 (37.01%) interviewers thought that gloves and masks were not necessary at work (Table 4).

**Table 4:** Concerns about zoonotic threats to humans.

**Questionnaires**	**Pig farmers (%)**	**Pig cutters (%)**	**Pig salesmen (%)**
**High risks of pigs/pork being infected/contaminated with zoonotic pathogens**			
Totally agree	66.77 (207/310)	15.53 (25/161)	10.94 (7/64)
Somewhat agree	29.35 (91/310)	58.36 (94/161)	51.56 (33/64)
Disagree	3.88 (12/310)	25.61 (42/161)	37.50 (24/64)
**High risks of zoonotic pathogens transmitted from pigs to humans**			
Totally agree	32.90 (102/310)	5.59 (9/161)	14.06 (9/64)
Somewhat agree	17.74 (55/310)	14.91 (24/161)	20.73 (18/64)
Disagree	49.35 (153/310)	79.50 (128/161)	57.81 (37/64)
**High risks of being exposed to infectious PRV viruses upon working**			
Totally agree	7.10 (22/310)	8.08 (13/161)	9.38 (6/64)
Somewhat agree	49.68 (154/310)	11.18 (18/161)	15.62 (10/64)
Disagree	43.22 (134/310)	80.74 (130/161)	75.00 (48/64)
**Major measures taken for the prevention of pseudorabies or other zoonosis upon working**			
Wearing gloves and mask	65.81 (204/310)	65.22 (105/161)	43.75 (28/64)
Dealing with wound in time	95.16 (295 /310)	90.06 (145/161)	89.06 (57/64)
Learning relevant knowledge	98.06 (304/310)	98.76 (159/161)	70.31 (42/64)

## Discussion

Recently, the potential cross-species transmission of PRV from pigs to humans has received extensive attention (5, 7, 8, 13). Indeed, these workers including pig farmers, pig cutters, and pork sellers are at high risk of being infected by PRV (14). Although the application of the various types of vaccines and other effective measures greatly prevent the occurrence of PR in China, PRV remains highly prevalent in China, which threatens the health of individuals, especially pig farmers and veterinarians (3).

In this study, we investigated the epidemiology of PRV in Hunan Province of China. Results showed that 1,667 serum samples (8.91%) and 38 tissue samples (2.33%) were positive for PRV antibodies and nucleic acids, respectively. The investigation results were significantly lower than a previous study conducted in Hunan Province (12). Two factors might contribute to this point: (1) since the outbreak of African swine fever (ASF) in 2018, pig farms in China, especially large-scale ones, have taken more strict control measures against ASF and other porcine diseases, including PRV, to ensure productivity; (2) in China, pork oversupply led to its price reduction recently, and many pig farms (especially large-scale pig farms) performed effective programs for the eradication of infectious diseases, including PR. It was worth noting that almost 20% of collected sites contained PRV-positive pig (s), which is consistent with the questionnaire survey results, indicative of a long way to go for the prevention of PRV. In addition, over 40% of pig farmers declared that they adopted live attenuated vaccines for PR control. Consequently, the infectious PRV might be widely distributed in the environment after vaccination (15), thereby posing a great threat to workers' lives. Thus, the potential threats of live attenuated PRV vaccines to humans should be concerned (16).

Many zoonotic diseases are major risks to pig farmers, especially, the occurrence of injury upon working allows their wounds to be exposed to the environment (17), such as Japanese encephalitis, Toxoplasmosis, and Streptococcosis (18–20). General self-protection behaviors and rapid wound treatment at work are very essential for preventing these zoonotic diseases. Worryingly, most of investigated workers declared that they did not form good self-protection habits upon working (such as wearing gloves, mask, etc.) and were often injured, even at the position of the head or face. Once PR occurred in pig farms, the infectious viruses would widely distribute in the environment, potentially rendering farmers to inhale the infectious viruses and their wounds or hands to contact with the viruses. Moreover, the frequent ignorance of clinical symptoms of PRV-positive fattening pigs undoubtedly makes the downstream workers including slaughter and sale staffs have the risk of being infected.

As the first survey on the knowledge of PRV among pig farmers, pig cutters, and pig salesmen in China. Our data and analysis indicated a general lack of understanding of this pathogen among these pig related practitioners, as demonstrated by the fact that almost all pig farmers and over half of pig cutters had an idea about PRV, while only a quarter of pig salesmen ever heard about it. The underlying reason for this discrepancy may derive from the requirement of pig farmers to prevent the pigs against viral infection including PRV, thereby allowing them to be more knowledgeable to this pathogen. Similarly, Zhou et al. (21) found that the knowledge of chicken farmers about Avian influenza was also higher than that of chicken vendors.

PRV can transmit from pigs to other susceptible animals and even humans through three main routes (vertical transmission, horizontal transmission and aerosol transmission) (15, 22). However, only a small minority of participants (especially pig cutters and salesmen) were aware of this, and most of them ignored the aerosol transmission route. In addition, over half of pig farmers and nearly all pig cutters and salesmen interviewed in this study were not aware that PRV could infect humans, and most of them did not know the clinical signs of PRV infection in humans. Given the serious lack of knowledge about PRV among these practitioners, especially pig cutters and salesmen, two aspects need to be emphasized: (1) the prevention of PRV infection often receives not enough attention; (2) In case these individuals suffer from PR, clinical signs like dyspnea, encephalitis and their pig-related career should firstly be identified, thereby reducing the possibility of misdiagnosis, delayed treatment, even death in hospital (23).

Zoonotic infections of individuals from pigs have raised widespread concern, especially in pig farms (24, 25). Likewise, we noticed the trepidation of some pig farmers concerning the high risks of being infected by zoonotic pathogens from pigs, and the requirement for effective preventive measures. Given this, we suggested that: (1) these workers should attach importance to self-protection upon working, such as wearing masks and gloves. (2) educational efforts should be made to raise the awareness of these workers concerning zoonotic pathogens which may transmit from pigs to humans, including PRV. (3) a routing screening for pathogens, in particular, potential zoonotic ones among breeding pigs, should be conducted, thereby reducing the risk of zoonotic infection among different workers.

## Conclusion

In conclusion, the present study revealed the persistent threat of PRV infection to the pig industry in China. Meanwhile, the survey indicated that pig-related practitioners had poor self-protection awareness upon working, and also lacked of associated knowledge about PRV, particularly those downstream workers like pig cutters and salesmen. Therefore, corresponding educational programs for these practitioners in terms of zoonotic pathogens including PRV should regularly be implemented.

## Data availability statement

The original contributions presented in the study are included in the article/Supplementary material, further inquiries can be directed to the corresponding author/s.

## Author contributions

AW, JY, YQ, YY, WW, and LT designed the experiments. YQ, YZ, LL, CW, PZ, DD, HL, NW, and LY contributed to the investigation and methodology. LT, JY, and AW contributed to writing—original draft preparation. LT, YQ,WW, and AW revised the manuscript. AW, JY, WW, and YY contributed in funding. All authors contributed to the article and approved the submitted version.

## Funding

This study was supported by the major specialized Projects of Yunan Science and Technology (No. 202102AE90007), the General Program of Natural Science Foundation of China (Grant No. 31571432/31802252), the National Key Research and Development Program of China (No. 2017YFD0501800), the Major Specialized Projects of Yunnan Science and Technology (No. 202102AE090007), the Hunan Provincial Natural Science Foundation of China (2020JJ4041), and the Special Fundamental Research Funding of Hunan Province Swine Industry Technology System. Supported was also provided by Furong Scholar funding to AW.

## Conflict of interest

Author YZ was employed by Hunan Sino-science Gene Technology Co., Ltd. Author AW was employed by PCB Biotechnology LLC. The remaining authors declare that the research was conducted in the absence of any commercial or financial relationships that could be construed as a potential conflict of interest.

## Publisher's note

All claims expressed in this article are solely those of the authors and do not necessarily represent those of their affiliated organizations, or those of the publisher, the editors and the reviewers. Any product that may be evaluated in this article, or claim that may be made by its manufacturer, is not guaranteed or endorsed by the publisher.
