# Supplementary material for: Pseudorabies in pig industry of China: Epidemiology in pigs and practitioner awareness
Source: Front Vet Sci. 2022 Sep 16;9:973450. doi: 10.3389/fvets.2022.973450 (PMC9536195; doi:10.3389/fvets.2022.973450)

**Supplementary Figure 1.**  Locations of two provinces in China where the questionnaire survey was mainly conducted.


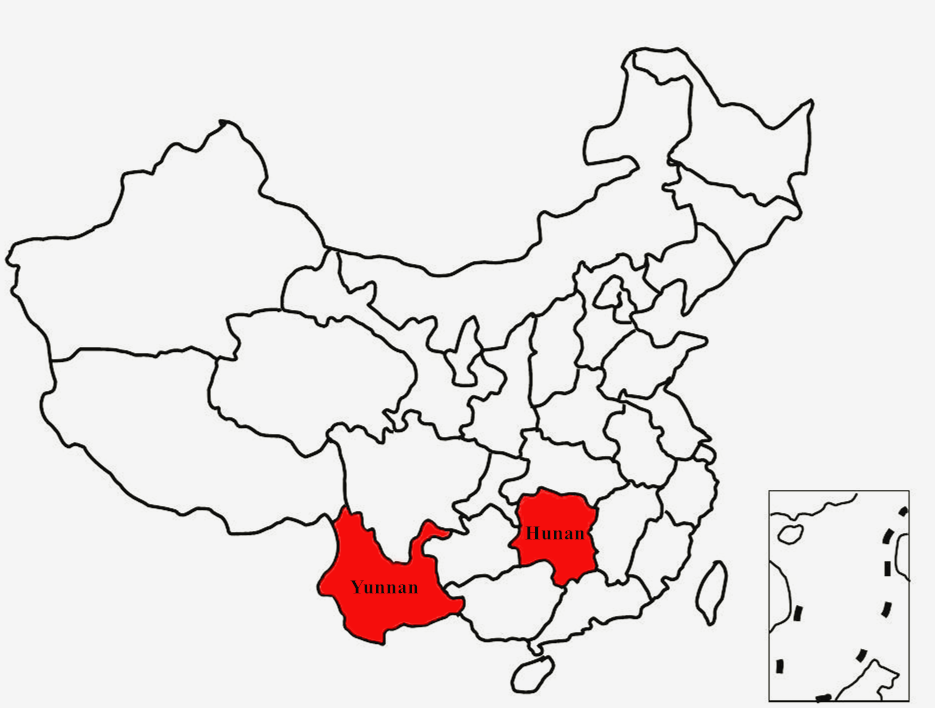

Supplement: Supplementary file 1 [file Table_1.DOC]
