# Supplementary material for: Pseudorabies in pig industry of China: Epidemiology in pigs and practitioner awareness
Source: Front Vet Sci. 2022 Sep 16;9:973450. doi: 10.3389/fvets.2022.973450 (PMC9536195; doi:10.3389/fvets.2022.973450)
Supplement: Supplementary file 2 [file Table_2.DOC]

**Supplementary Table 1** Main characteristics of the questionnaire respondents

| **Characteristics** | **Pig farmers (n=310)** | **%** | **Pig cutters (n=161)** | **%** | **Pork salesmen (n=64)** | **%** |
| --- | --- | --- | --- | --- | --- | --- |
| **Gender** | | | | | | |
| Male | 220 | 70.97 | 113 | 70.19 | 43 | 67.19 |
| Female | 90 | 29.03 | 48 | 29.81 | 21 | 22.81 |
| **Age group** | | | | | | |
| <20 | 10 | 3.23 | 3 | 1.86 | NA | NA |
| 20~30 | 157 | 50.65 | 39 | 24.22 | NA | NA |
| 30~40 | 77 | 24.84 | 49 | 30.43 | 15 | 23.44 |
| 40~50 | 45 | 14.52 | 41 | 25.47 | 17 | 26.56 |
| >50 | 21 | 6.77 | 29 | 18.02 | 32 | 50.0 |
| **Educational level** | | | | | | |
| Primary school or below | 8 | 2.58 | 9 | 5.59 | 5 | 7.81 |
| Secondary school | 59 | 19.03 | 67 | 41.62 | 24 | 37.50 |
| High school | 152 | 49.03 | 58 | 36.02 | 35 | 56.69 |
| Bachelor degree | 64 | 20.65 | 22 | 13.66 | NA | NA |
| Master’s degree or above | 27 | 8.71 | 5 | 3.11 | NA | NA |
| **Breeding scale** | | | | | | |
| Free range farm | 38 | 12.26 | **-** | **-** | **-** | **-** |
| Small size farm | 55 | 17.74 | **-** | **-** | **-** | **-** |
| Medium size farm | 112 | 36.13 | **-** | **-** | **-** | **-** |
| Large size farm | 81 | 26.13 | **-** | **-** | **-** | **-** |
| Breeding farm | 24 | 7.74 | **-** | **-** | **-** | **-** |
| **Position** | | | | | | |
| Assistant farmer or farm manager | 45 | 14.52 | **-** | **-** | **-** | **-** |
| Breeder supervisor | 52 | 16.77 | **-** | **-** | **-** | **-** |
| Veterinarian | 34 | 10.97 | **-** | **-** | **-** | **-** |
| Breeder | 179 | 55.74 | **-** | **-** | **-** | **-** |
| **Working year** | | | | | | |
| <2 years | 117 | 37.74 | 26 | 16.15 | NA | NA |
| 2~6 years | 81 | 26.13 | 51 | 31.68 | NA | NA |
| 6~10 years | 39 | 12.58 | 30 | 18.63 | 10 | 15.63 |
| 10~20 years | 52 | 16.77 | 27 | 16.77 | 29 | 45.31 |
| >20 years | 21 | 6.78 | 27 | 16.77 | 25 | 39.06 |
| **Working region** | | | | | | |
| Hunan | 145 | 46.77 | 98 | 60.87 | 42 | 65.63 |
| Yunnan | 78 | 25.16 | 59 | 36.65 | 22 | 34.37 |
| Hebei | 38 | 12.26 | NA | NA | NA | NA |
| Others | 49 | 15.81 | 4 | 2.48 | NA | NA |
